# Epithelial-Mesenchymal Transitional Markers in Endometrial Carcinoma: A Review of Clinicopathological Correlation

**DOI:** 10.7759/cureus.110638

**Published:** 2026-06-11

**Authors:** M Kalaivani, Divya Dhanabal, Leena Dennis Joseph

**Affiliations:** 1 Department of Pathology, Sri Ramachandra Institute of Higher Education and Research, Chennai, IND

**Keywords:** epithelial-mesenchymal transition, reproductive health, slug, snail, twist, zeb2

## Abstract

Endometrial carcinoma is the most frequently diagnosed gynaecological malignancy in developed countries, and its global incidence continues to increase. Myometrial invasion and lymphovascular spread, driven by epithelial-mesenchymal transition (EMT), are among the most important determinants of clinical outcome. EMT is a dynamic biological process in which epithelial cells develop migratory and invasive mesenchymal characteristics under the influence of transcription factors including SNAIL (SNAI1), SLUG (SNAI2), TWIST, and ZEB2. The aims of this review are to review the current evidence on the immunohistochemical (IHC) expression of the four important EMT transcription factors (SLUG, TWIST, SNAIL, and ZEB2) in endometrial carcinoma and to focus on their clinicopathological correlations, prognostic implications, signalling mechanisms, and potential as therapeutic targets. We conducted a narrative review of the published literature in English using the PubMed, Scopus, and Google Scholar databases. We retrieved and reviewed articles on IHC or molecular expression of SLUG, TWIST, SNAIL, and ZEB2 in endometrial carcinoma. SLUG overexpression independently predicted the advanced International Federation of Gynecology and Obstetrics (FIGO) stage, deep myometrial invasion, lymphovascular space invasion (LVSI), and poorer five-year survival. TWIST expression showed a significant association with the depth of myometrial invasion and loss of E-cadherin expression and was also found to be an independent prognostic factor in multivariate analysis. SNAIL correlates with histological grade, peritoneal cytology positivity, and non-endometrioid histology. High levels of ZEB2 are related to adnexal involvement and higher FIGO stage. Together, these four markers represent a prognostically relevant panel of markers reflecting the invasive phenotype of endometrial carcinoma, which might be targets for novel therapeutic intervention.

## Introduction and background

Endometrial carcinoma (EC) is the most frequently diagnosed malignant neoplasm of the female genital tract in developed nations. It ranks as the fourth most common cancer among women worldwide. Although most cases are identified at an early stage that can be treated surgically, a significant subset presents with or subsequently develops myometrial invasion, lymph node metastasis, and distant spread, which substantially worsen prognosis. The five-year survival for stage I disease approaches 90% but drops to below 20% for stage IV, underscoring the critical importance of elucidating the mechanisms that drive tumour progression [1].

Conventional histopathological parameters such as tumour grade, depth of myometrial invasion, the International Federation of Gynecology and Obstetrics (FIGO) stage, lymphovascular space invasion (LVSI), and cervical involvement are commonly used to guide decisions regarding adjuvant therapy. However, these factors do not completely represent the tumour's biological behaviour. Recent advances have demonstrated that immunohistochemical (IHC) and molecular markers, including p53 status, mismatch repair (MMR) protein expression, and DNA polymerase epsilon (POLE) mutations, provide additional prognostic information and enable more precise risk stratification of ECs. Increasing evidence suggests that epithelial-mesenchymal transition (EMT) plays a key molecular role in myometrial invasion, peritoneal dissemination, and the eventual metastatic spread of EC.

EMT is a highly conserved and dynamic cellular process initially identified during embryogenesis, in which epithelial cells lose their polarity and intercellular adhesion, acquire mesenchymal characteristics, and develop migratory and invasive properties. In cancer biology, this process, referred to as type 3 EMT, enables tumour cells to detach from the basement membrane, invade the surrounding stroma, enter the circulation, and form metastatic lesions at distant sites. A key molecular event in EMT is the transcriptional suppression of E-cadherin, a transmembrane glycoprotein essential for maintaining adherens junctions and epithelial integrity. This suppression is regulated by multiple families of transcription factors, primarily the Snail family (SNAIL and SLUG), the basic helix-loop-helix (bHLH) family (TWIST), and the zinc finger E-box binding homeobox family, which includes ZEB1 and ZEB2 [2].

IHC serves as an accessible and effective method for assessing the expression of EMT-related transcription factors in routinely processed formalin-fixed paraffin-embedded (FFPE) tumour specimens. IHC-based assessment relates protein expression to histomorphological context and clinicopathological parameters, opening up the possibility of research inquiry and possible clinical application. This review will focus on the IHC expression of SLUG, TWIST, SNAIL, and ZEB2 in EC, critically reviewing the published data on their expression patterns, clinicopathological associations, prognostic significance, mechanistic involvement, and therapeutic implications.

## Review

Methodology

A literature search was conducted using the PubMed, Scopus, and Google Scholar databases from database inception to May 2026. The final search was performed on 15 May 2026. Search terms included combinations of "endometrial carcinoma", "endometrial cancer", "epithelial-mesenchymal transition", "EMT", "SNAIL", "SLUG", "TWIST", "ZEB1", "ZEB2", "immunohistochemistry", and "prognosis" using Boolean operators. English language articles relevant to the molecular, pathological, prognostic, and therapeutic aspects of EMT in EC were reviewed. Additional articles were identified through cross-referencing of bibliographies. Articles were included if they evaluated EMT in EC, with particular emphasis on EMT-related molecular alterations, IHC markers, clinicopathological correlations, prognostic significance, and therapeutic implications. Original research articles, review articles, and relevant experimental studies published in English were considered. Studies unrelated to EC, non-English publications, duplicate reports, and articles lacking sufficient information on EMT-related pathways or biomarkers were excluded. As this was a narrative review, no formal meta-analysis or risk-of-bias assessment was undertaken.

Overview of EMT

Definition and Classification

EMT was first conceptualised in the 1980s in studies of embryonic morphogenesis by Elizabeth Hay. Kalluri and Weinberg later classified it into three different subtypes. Type 1 EMT plays an important role in implantation, embryonic development, and organogenesis. Type 2 EMT is associated with wound healing, tissue repair, fibrosis, and regeneration. Type 3 EMT is related to cancer progression and facilitates the invasion and metastasis of neoplastic epithelial cells [3,4].

EMT is a process involving profound morphological and molecular changes in cells. These cells lose their apical-basal polarity, while tight junctions and desmosomes become disassembled. Epithelial markers such as E-cadherin, cytokeratins, and occludin are downregulated, whereas mesenchymal markers including alpha-smooth muscle actin (α-SMA), fibronectin, vimentin, and N-cadherin are upregulated. Simultaneous induction of matrix metalloproteinases (MMPs) also allows for extracellular matrix degradation and tissue infiltration [3].

Molecular Mechanisms of EMT Transcription Factors

The transcriptional reprogramming that drives EMT is controlled by three major families of transcription factors that directly bind and repress the CDH1 promoter (encoding E-cadherin) and co-regulate a broad network of epithelial and mesenchymal genes:

Snail family (SNAI1/SNAIL and SNAI2/SLUG) zinc finger transcriptional repressors recognise E-box sequences (5'-CAGGTG-3') within promoters of target genes. SNAIL was initially discovered as a regulator of mesoderm development in *Drosophila melanogaster* [5]. SLUG, also known as SNAI2, is a structural homologue of SNAIL but differs in expression patterns, post-translational regulation, and functional specificity in different types of cancer.

The bHLH transcription factors TWIST1 and TWIST2, which bind to E-box sequences in target gene promoters and form homo- or heterodimeric complexes via the HLH domain, make up the TWIST family. TWIST proteins promote the expression of N-cadherin, which accelerates the cadherin switch and increases invasiveness while suppressing the expression of E-cadherin [6].

With two zinc finger domains encircling a central homeodomain, the ZEB family (ZEB1/TCF8 and ZEB2/SIP1) binds E-box domains in the CDH1 promoter with a high degree of specificity. The miR-200 family and ZEB proteins are involved in a double-negative feedback loop. The miR-200 family suppresses the expression of ZEB1 and ZEB2, whereas ZEB proteins inhibit miR-200 expression. This reciprocal regulation helps determine whether cells maintain an epithelial phenotype or acquire a mesenchymal state [7].

Signalling Pathways Inducing EMT in Endometrial Cancer

In the endometrial cancer microenvironment, multiple extracellular signals converge to activate EMT transcription factors. The most potent EMT inducer in epithelial cells is transforming growth factor-beta (TGF-β), and its downstream Smad signalling directly increases the expression of SNAIL, SLUG, and ZEB. Wnt/β-catenin signalling stabilises and translocates β-catenin to the nucleus to stimulate SLUG and other EMT effector transcription. SNAIL and SLUG are also upregulated by the phosphoinositide 3-kinase/protein kinase B/mechanistic target of rapamycin (PI3K/AKT/mTOR) pathway, which is often dysregulated by loss of phosphatase and tensin homolog (PTEN) in endometrioid EC [3]. Oestrogen is a major driver of type 1 endometrial carcinogenesis and has been shown to induce EMT in endometrial cancer cell lines through PI3K and extracellular signal-regulated kinase (ERK) signalling, increasing SNAIL, SLUG, and ZEB2 expression and decreasing E-cadherin. Notch, Hedgehog, vascular endothelial growth factor (VEGF), epidermal growth factor (EGF), and platelet-derived growth factor (PDGF) signalling pathways also promote EMT in EC. In addition, tumour-associated stromal components, such as cancer-associated fibroblasts, contribute to tumour growth and EMT activation in different histological and molecular subtypes of the tumour [1].

The specific association of EMT with endometrioid EC with the microcystic, elongated, and fragmented (MELF) pattern of myometrial invasion has been demonstrated [1,8]. IHC showed strong CK7 expression, decreased E-cadherin and hormone receptor expression, and increased SNAIL expression at the invasive front, providing histomorphologic support for EMT-driven invasion in routine surgical pathology specimens.

SNAIL (SNAI1) in endometrial cancer

Mechanism and Architecture

The zinc finger transcriptional repressor SNAIL (SNAI1) directly attaches to E-box sequences in the CDH1 promoter region and recruits the switch-independent 3a/histone deacetylase 1/histone deacetylase 2 (Sin3A/HDAC1/HDAC2) complex, leading to epigenetic silencing of the gene. It also downregulates the expression of claudins, occludin, and other tight junction proteins, thus compromising epithelial integrity in a comprehensive way. SNAIL expression is tightly regulated at the post-translational level: glycogen synthase kinase-3 beta (GSK-3β)-mediated phosphorylation promotes SNAIL nuclear export and proteasomal degradation, while PI3K/AKT signalling inhibits GSK-3β, to stabilise SNAIL protein and allow sustained EMT [9].

IHC Expression in Endometrial Cancer

IHC has been widely used to study SNAIL in tissue specimens of EC. Tanaka et al. demonstrated the nuclear expression of SNAIL in 16.9% of primary tumours in an IHC analysis of 354 primary endometrial tumours by tissue microarray (TMA). Histological type, depth of myometrial invasion, FIGO stage, positive peritoneal cytology, and survival rate for patients were all significantly correlated with nuclear SNAIL expression and low E-cadherin. Nuclear SNAIL expression and low E-cadherin are independent predictors of progression-free and overall survival [10].

Sadłecki et al. performed an IHC study on 158 surgically removed EC specimens from Polish patients and reported nuclear SNAIL positivity in 84.5% of cases. The total positivity rate of SNAIL was high, but its expression was not statistically correlated with each clinicopathological variable, such as myometrial invasion depth, cervical invasion, LVSI, lymph node status, and FIGO stage, in this cohort. This can be the role of SNAIL as an early but transient EMT inducer rather than a persistent driver of advanced disease [2].

Blechschmidt et al. demonstrated that SNAIL expression at the myoinvasive front of endometrioid adenocarcinomas inversely correlates with E-cadherin immunoreactivity. This explains the role of SNAIL in the functional transcriptional repression of E-cadherin at the invasive interface. The inverse association was confirmed by several follow-up studies [11].

Abouhashem et al. demonstrated that the combined assessment of hypoxia-inducible factor 1α (HIF-1α), SNAIL, and E-cadherin showed an HIF-1α/SNAIL/E-cadherin axis in endometrioid EC, suggesting that intratumoural hypoxia promotes SNAIL-dependent EMT, particularly at the invasive tumour margins [12].

Clinicopathologic Correlations

SNAIL expression is invariably associated with the following: (a) non-endometrioid histological type, particularly uterine serous carcinoma and clear cell carcinoma; (b) higher histological grade (G3 > G2 > G1); (c) deep myometrial invasion (≥50%); (d) positive peritoneal cytology; and (e) overall shorter patient survival. In endometrial cancer, high SNAIL expression is linked to reduced oestrogen receptor alpha (ERα) expression, making the cancer hormone-independent. This hormone independence is associated with a more aggressive cancer phenotype. SNAIL also drives EMT, and together these effects form a consistent mechanistic model connecting hormone independence, aggressive behaviour, and EMT induction.

SLUG (SNAI2) in EC

Design and Operation

The SNAI2 gene encodes a SNAIL paralogue called SLUG (SNAI2). It shares the same structure as the C-terminal zinc finger domain but differs significantly in its N-terminal SNAG (Snail/Gfi repressor) domain regulation and downstream targets. In contrast to SNAIL, SLUG is more stable in EMT-active cells and is highly expressed in basal-like and mesenchymal-type cancer cells. SLUG represses E-cadherin and directly inhibits pro-apoptotic proteins PUMA and BIM, blocking apoptotic pathways that are both p53-dependent and p53-independent. This makes cancer cells resistant to both genotoxic and intrinsic apoptotic stimuli, thereby promoting cancer cell survival. SLUG-expressing tumour cells get a double benefit from their anti-apoptotic function, increasing their invasiveness and their ability to resist cell death signals [13].

IHC Expression

SLUG is the most clinically relevant EMT transcription factor in EC among the four EMT transcription factors discussed here. In the cohort of 158 EC patients, Sadłecki et al. found the highest positivity among all tested transcription factors for nuclear SLUG positivity in 92.2% of specimens. Notably, SLUG expression was significantly upregulated in patients with FIGO stage III/IV disease, type 2 EC (non-endometrioid histology), myometrial invasion ≥50%, adnexal involvement, and distant metastases [2]. Univariate analysis revealed that SLUG was significantly associated with advanced FIGO stage. SLUG overexpression was found to be a significant predictor of five-year mortality by univariate as well as multivariate survival analyses. Each unit increase in nuclear SLUG expression was associated with a substantial increase in the mortality hazard ratio [2].

In Tanaka et al.'s TMA study of 354 cases, nuclear SLUG positivity was found in 3.7% of primary tumours. The much lower frequency of nuclear positive tumours is likely due to their strict criteria for scoring nuclear positivity, in contrast to the broad positive threshold used in the Polish cohort. SLUG expression was significantly correlated with LVSI, lymph node metastases, and recurrence [10]. Differences in reported positivity rates between the studies highlight the need for standardisation of antibody selection, antigen retrieval, and scoring methods in IHC studies of EMT markers.

Chemoresistance and SLUG

The role of SLUG in mediating therapeutic resistance has significant clinical implications. Cancer cells overexpressing SLUG are resistant to cytotoxic chemotherapy (carboplatin, paclitaxel) and radiation therapy by suppressing apoptotic pathways and conferring stem cell-like properties [14]. Moreover, SLUG expression is associated with the cancer stem cell (CSC) phenotype characterised by self-renewal, tumour-initiating capacity, and the intrinsic upregulation of drug efflux pumps. SLUG expression in the primary tumour may be used as a marker to identify individuals who need more intensive adjuvant therapy or new targeted methods, since patients with extrauterine disease at diagnosis have micrometastases and are more likely to recur.

EC with TWIST1 expression

Structure and Function

The bHLH transcription factor TWIST1 (also known as TWIST) detects E-box sequences and acts as a dimer. It mediates the suppression of E-cadherin and causes the cadherin switch, which is the downregulation of E-cadherin and the overexpression of N-cadherin, as well as a broad transcriptional program that is orientated toward angiogenesis, anoikis resistance, and cell migration [15]. TWIST1 induces cell motility via the upregulation of fibronectin and vimentin, downregulation of E-cadherin, and activation of AKT2/integrin β1 signalling axis. Nuclear factor kappa B (NF-κB)-mediated upregulation of TWIST-1, a mechanism supported by studies demonstrating NF-κB/miRNA regulatory feedback loops in chemotherapy-induced EMT, leads to resistance development in tumour cells to cytotoxic drugs such as paclitaxel and doxorubicin [16,17]. The paralogue TWIST2 has different expression characteristics: in EC, TWIST2 immunoexpression is cytoplasmic, not nuclear, and therefore, interpretation of IHC results depends on the marker and protocol [2].

Selected Studies on IHC Expression

TWIST expression by IHC in 70 endometrial cancer specimens was reported in a study by Kyo et al. Using a semi-quantitative system considering staining intensity and extent, they scored 36 cases (51%) as high TWIST expression and 34 (49%) as low. TWIST staining was cytoplasmic and nuclear and was mostly observed in cancer cell nests, especially around the tumour's invasive margin. Some adjacent stromal cells also showed positive results. Deep myometrial invasion and the concurrent loss of E-cadherin had a strong correlation with high TWIST expression. Additionally, TWIST was found to be a greater independent prognostic factor than myometrial invasion and an independent predictor of patients' overall survival by multivariate Cox regression analysis [15].

In the large cohort study by Sadłecki et al., TWIST1 IHC positivity was detected in 10.9% of the specimens, whereas TWIST2 was universally positive (100%) and located in the cytoplasm. The striking difference in the expression pattern between the two TWIST paralogues (nuclear TWIST1 in a minority of cases and ubiquitous cytoplasmic TWIST2) suggests that the two proteins may have non-redundant roles in endometrial carcinogenesis and that cytoplasmic TWIST2 could be a marker of post-translational modifications. In this cohort, neither TWIST1 nor TWIST2 was statistically significantly associated with any of the individual clinicopathological variables [2].

Feng et al. used IHC and TMA in a series of 124 endometrioid ECs and showed that HIF-1α, TWIST, and E-cadherin form a coordinated pathway, where hypoxic induction of HIF-1α leads to TWIST overexpression, which then suppresses E-cadherin. Both HIF-1α and TWIST were significantly increased in tumours with higher grade, higher stage, and deep myometrial invasion compared with normal endometrium and atypical hyperplasia. This supports the concept of HIF-1α/TWIST co-activation in aggressive endometrioid EC [18].

TWIST expression was higher in G3 tumours and deep myometrial invasion (>1/2 thickness) compared with G2 tumours and superficially invasive carcinomas, supporting a role in aggressive invasion. The association between TWIST-positive endometrial cancer cells and tumour-associated macrophages (TAMs) has been studied recently. These studies demonstrated that TAMs can promote EMT by activating TWIST through the epidermal growth factor receptor (EGFR)/ERK1/2 signalling pathway. This finding links the immune microenvironment to EMT-mediated invasion in endometrial cancer [19].

TWIST and Clinical Relevance

TWIST is an independent predictive factor for endometrial cancer, according to multivariate survival analysis. It also showed that IHC assessment of TWIST expression in surgical specimens may be used to support existing pathologic staging to identify high-risk patients. TWIST1 was studied by Shen et al. in a retrospective cohort of type 1 endometrial cancer and premalignant lesions. Their expression showed a gradual increase from normal endometrium to atypical hyperplasia and finally to invasive carcinoma, indicating their possible role as markers of neoplastic transformation and prognostic indicators [6].

ZEB2 in endometrial cancer

Structure and Mechanism

Zinc finger E-box binding homeobox family member ZEB2, also referred to as SMAD-interacting protein 1 (SIP1), is encoded by the ZEB2 gene. ZEB2 contains zinc finger clusters at both the N- and C-terminal regions, along with a central homeodomain and a SMAD-binding domain. These structural features enable it to interact with R-SMAD proteins downstream of the TGF-β signalling pathway. This structural arrangement connects TGF-β growth factor signalling to the transcriptional control of EMT [20]. ZEB2 binds to E-box sequences to repress E-cadherin and also represses other components of the epithelial junctional complex, such as plakophilin-2, desmoplakin, and occludin. ZEB2 activity is tightly regulated by the miR-200 family. miR-200 family members silence ZEB2 post-transcriptionally by targeting its 3'-UTR, while ZEB2 protein represses miR-200 promoters transcriptionally. This sets up a bistable double-negative feedback loop, which locks either the epithelial or mesenchymal cell state. Epigenetic silencing of miR-200 or ZEB2 amplification tips the balance and locks the cells in the mesenchymal invasive state [21].

Significant Studies on IHC Expression

The positivity rate of ZEB2 in the EC tissue was high. Sadłecki et al. reported that 98% of EC specimens were ZEB2 positive by IHC, with immunoexpression found in the cytoplasm. This cytoplasmic pattern is likely due to the post-translational regulation of ZEB2 and association with cytoplasmic SMAD complexes. Patients with adnexal involvement had considerably higher ZEB2 expression than patients without adnexal involvement. On univariate regression analysis, the combination of SLUG and ZEB2 was a strong predictor of advanced FIGO staging (stage III/IV) [2].

Wong et al. reported the role of ZEB2 as an E-cadherin repressor and its overexpression in several cancers. Later studies in endometrial cancer demonstrated that the ZEB2/miR-200 axis is functionally active with decreased miR-200 expression correlating with ZEB2 overexpression and enhanced migration and invasion in EC cell line models. A 2022 Frontiers in Oncology review on endocrine-EMT intersections in EC reported that oestradiol treatment upregulates SNAIL, SLUG, and ZEB2 expression through PI3K and ERK signalling pathways. This directly links hormonal stimulation to ZEB2-mediated EMT in endometrial cancer [22].

Also, overexpression of ZEB2 has been linked with advanced nodal stage, distant metastasis, and poor survival in gynaecologic and other epithelial malignancies. Its value as a predictor of adnexal involvement in EC is of particular importance as adnexal spread is stage IIIA disease and significantly alters adjuvant therapy planning [2].

miR-200/ZEB2 Axis and Stem Cells in Cancer

Both the regulation of EMT and the preservation of the characteristics of CSCs depend heavily on the miR-200/ZEB axis. ZEB2 overexpression induces a stem-like cell state with self-renewal, tumour initiation, and chemotherapy resistance properties via the repression of miR-200. This link between ZEB2, EMT, and CSC biology in endometrial cancer has profound implications for understanding tumour recurrence and treatment failure, especially in high-grade and non-endometrioid subtypes [23].

Clinicopathological correlation and comparative analysis

A comprehensive review of the four EMT transcription factors in published IHC studies provides several key insights with both research and clinical implications in EC.

Almost all ECs are positive for TWIST2 (100%) and ZEB2 (98%) by IHC, and SLUG (92.2%) and SNAIL (84.5%) are also very frequent. In contrast, TWIST1 is less positive (~10.9-51% depending on study and scoring methodology), indicating that its activation may be more context-dependent or a later event in EC progression in some histological subtypes. This large variability of positivity rates of the same marker in different studies supports the need for methodological standardisation in future studies [2].

SLUG was the most powerful independent predictor of unfavourable clinicopathological parameters, including advanced stage, deep myometrial invasion, adnexal involvement, distant metastasis, and five-year survival [23]. Multivariate survival analysis identified TWIST1 as the most important independent prognostic factor. SNAIL showed the strongest correlation with loss of E-cadherin and with histological grade at the invasive front. ZEB2 was most specifically associated with adnexal spread and predicted advanced FIGO stage, together with SLUG [2].

Regarding the subcellular localisation, SNAIL, SLUG, and TWIST1 show a predominantly nuclear expression, which is consistent with the fact that they are active transcriptional repressors in the nucleus. TWIST2 and ZEB2 are localised in the cytoplasm, which may indicate specific post-translational modifications, protein-protein interactions (e.g., ZEB2 with SMAD proteins), or alternative isoforms [2]. Correct subcellular localisation is important when interpreting IHC for these markers, with nuclear positivity generally reflecting active transcriptional activity.

Among the EMT-related biomarkers studied in EC, SLUG appears to be the most important prognostic marker, followed by TWIST, while SNAIL and ZEB2 seem to play a roughly equal role. SLUG is the only marker to demonstrate independent multivariate prognostic value for survival in a large dedicated study [2]. The most comprehensive risk stratification is most likely achieved with a combined EMT panel including all four markers, E-cadherin, and N-cadherin.

Technical aspects of IHC for EMT markers

The standardisation of IHC protocols is of utmost importance for the reliable assessment of EMT transcription factors expressed at different levels and in different subcellular compartments. The following technical issues are of importance when evaluating SLUG, TWIST, SNAIL, and ZEB2 in FFPE EC tissue:

Antibody Selection

Rabbit polyclonal anti-SNAIL antibodies (e.g., NBP1-80022, Novus Biologicals, Centennial, CO, USA; 1:100 dilution), mouse monoclonal anti-SLUG antibodies (e.g., NBP2-03886, Novus Biologicals, Centennial, CO, USA; 1:200 dilution), mouse monoclonal anti-TWIST1 antibodies (e.g., ab50887, Abcam, Cambridge, UK; 1:20 dilution), and rabbit polyclonal anti-ZEB2 antibodies (e.g., HPA003456, Sigma-Aldrich, Burlington, MA, USA; 1:100 dilution) have been utilised in published studies with incubation times of 30-60 minutes. Validation of antibody specificity is essential and requires positive and negative controls. Standardised platforms with validated clones are required for inter-laboratory comparability [2].

Scoring Systems

There are two major approaches. The first is an assessment of nuclear/cytoplasmic positivity (proportion of positive cells and staining intensity, expressed as H-scores or Allred scores). The second is semi-quantitative scoring, which combines intensity (0-3) and extent (0-4) to give a composite score. For studies on TWIST specifically, a nuclear scoring system based on extent (0-3) and intensity (0-3) has been used. Cut-points for "high" vs "low" expression were derived using receiver operating characteristic (ROC) curve analysis or median values [2,23,24]. Meta-analytic synthesis would be possible if a validated H-score system were used uniformly across centres.

Controls and Interpretation

Adequate positive controls (e.g., invasive breast carcinoma or colon carcinoma for SNAIL and SLUG, embryonal rhabdomyosarcoma for TWIST, ovarian carcinoma for ZEB2) and negative controls (isotype antibody substitution) are needed for each staining run. Reference points for intensity calibration can be the intrinsic controls in EC sections, such as staining of myometrial smooth muscle cells, stromal fibroblasts, and blood vessel endothelium. Automated immunostaining platforms (e.g., Dako Autostainer Link 48; Ventana BenchMark ULTRA, Ventana Medical Systems, Inc., Tucson, AZ, USA) offer better reproducibility for the assessment of EMT markers, as manual staining results in inter-technician variability. Automated platforms with standardised polymer-based detection systems (EnVision FLEX, OptiView) and 3,3'-diaminobenzidine (DAB) chromogen would benefit studies across multiple institutions.

Therapeutic implications

Overexpression of SLUG, TWIST, SNAIL, and ZEB2 is strongly associated with aggressive tumour behaviour, chemoresistance, and poor outcome in EC. This motivates the investigation of these factors as therapeutic targets. There are some strategies being considered:

Inhibition of the TGF-β Pathway

TGF-β acts as a major upstream regulator of the four EMT transcription factors. Inhibition of TGF-β receptor signalling using small-molecule inhibitors such as galunisertib (LY2157299) or neutralising antibodies is currently being explored in gynaecologic as well as other solid tumours. Inhibition of TGF-β may reverse EMT, restore E-cadherin expression, reduce invasiveness, and potentially re-sensitise tumours to conventional therapies [25].

Reintroduction of miR-200

The miR-200 family inhibits ZEB2 (and ZEB1). Hence, restoration of miR-200 by miRNA mimics or nanoparticle delivery systems may be a promising approach to inhibit ZEB-driven EMT in endometrial and ovarian cancers [23,26]. Preclinical studies have demonstrated that forced expression of miR-200c reduces invasion, migration, and tumour growth in EC cell lines [26].

Epigenetic Therapies

SNAIL silences E-cadherin by recruiting HDAC1/2, and this repression can be reversed using HDAC inhibitors such as vorinostat and entinostat. By de-repressing E-cadherin transcription, these inhibitors have the potential to reverse the mesenchymal state of cancer cells. HDAC inhibitors in combination with the standard platinum-based chemotherapy are being investigated in clinical trials for recurrent endometrial cancer [27,28].

Inhibition of PI3K/AKT/mTOR

PI3K/AKT signalling stabilises SNAIL and SLUG and prevents their GSK-3β-mediated degradation. Therefore, clinically used PI3K inhibitors and mTOR inhibitors (temsirolimus, everolimus) for EC may have partial anti-EMT effects by destabilising SNAIL/SLUG, a secondary benefit that should be systematically investigated [9,29].

Targeting CSCs

Both SLUG and ZEB2 promote CSC identity. The EMT process may be indirectly inhibited by therapies targeting CSC surface markers (cluster of differentiation 44 (CD44), aldehyde dehydrogenase 1 (ALDH1)) or CSC signalling (Notch, Hedgehog). Salinomycin is an ionophore antibiotic with selective cytotoxicity against CSCs and was effective in preclinical models against endometrial CSC-like cells expressing SLUG [30,31]. Beyond directly targeting EMT pathways, researchers have also turned their attention to cancer cell metabolism, particularly the mitochondrial pathways that control apoptosis. Disrupting these metabolic processes is being explored as a complementary strategy to help overcome the treatment resistance that EMT drives in cancer cells.

## Conclusions

The four EMT transcription factors discussed in this review, SLUG, TWIST, SNAIL, and ZEB2, together comprise a biologically consistent and clinically relevant panel to define invasive behaviour in EC. Their IHC evaluation in routine surgical specimens provides a practical link between molecular biology and clinical pathology.

SLUG is the most powerful prognostic marker. Overexpression of SLUG is an independent predictor for poor five-year survival and is associated with several poor clinicopathological features. TWIST has been identified as an independent predictor of survival and may be included in prognostic panels along with conventional prognostic factors. SNAIL, a prototype E-cadherin repressor, plays an important role in the pathological evaluation of invasive margins and detection of the MELF pattern. It also has a well-established association with myometrial invasion. ZEB2 acts at the intersection of EMT and cancer stemness through the miR-200/ZEB axis. Therefore, it is considered a promising target for future therapy.

Future work should include (1) large multicentric prospective studies using standardised IHC protocols to determine reliable cut-off values and prognostic thresholds for each marker; (2) the integration of EMT transcription factor expression with the four-tier molecular classification of EC (POLE-ultramutated, microsatellite instability (MSI)-high/mismatch repair deficiency (MMRd), copy-number-high/TP53-mutant, and no specific molecular profile (NSMP)); (3) the development of a validated, clinically applicable IHC panel with the combination of SLUG, TWIST, SNAIL, ZEB2, and E-cadherin as a composite EMT score; (4) the exploration of liquid biopsy approaches to detect circulating EMT marker-expressing tumour cells; and (5) clinical trials evaluating the targeted inhibition of the TGF-β/SNAIL/SLUG axis or miR-200 restoration in recurrent and metastatic EC.

Translational research on EMT markers is emerging as a promising area in the management of endometrial cancer. It may help improve prognostic stratification, guide individualised adjuvant therapy, and improve patient survival.
